# Supplementary material for: Causal Relationship between Aging and Anorexia Nervosa: A White-Matter-Microstructure-Mediated Mendelian Randomization Analysis
Source: Biomedicines. 2024 Aug 16;12(8):1874. doi: 10.3390/biomedicines12081874 (PMC11351342; doi:10.3390/biomedicines12081874)
Supplement: Supplementary file 1 [file biomedicines-12-01874-s001.zip › Supplementary Figure/Figure S9/Leaveoneout plot of MR analysis for ubm-b-1920 to AN.pdf]

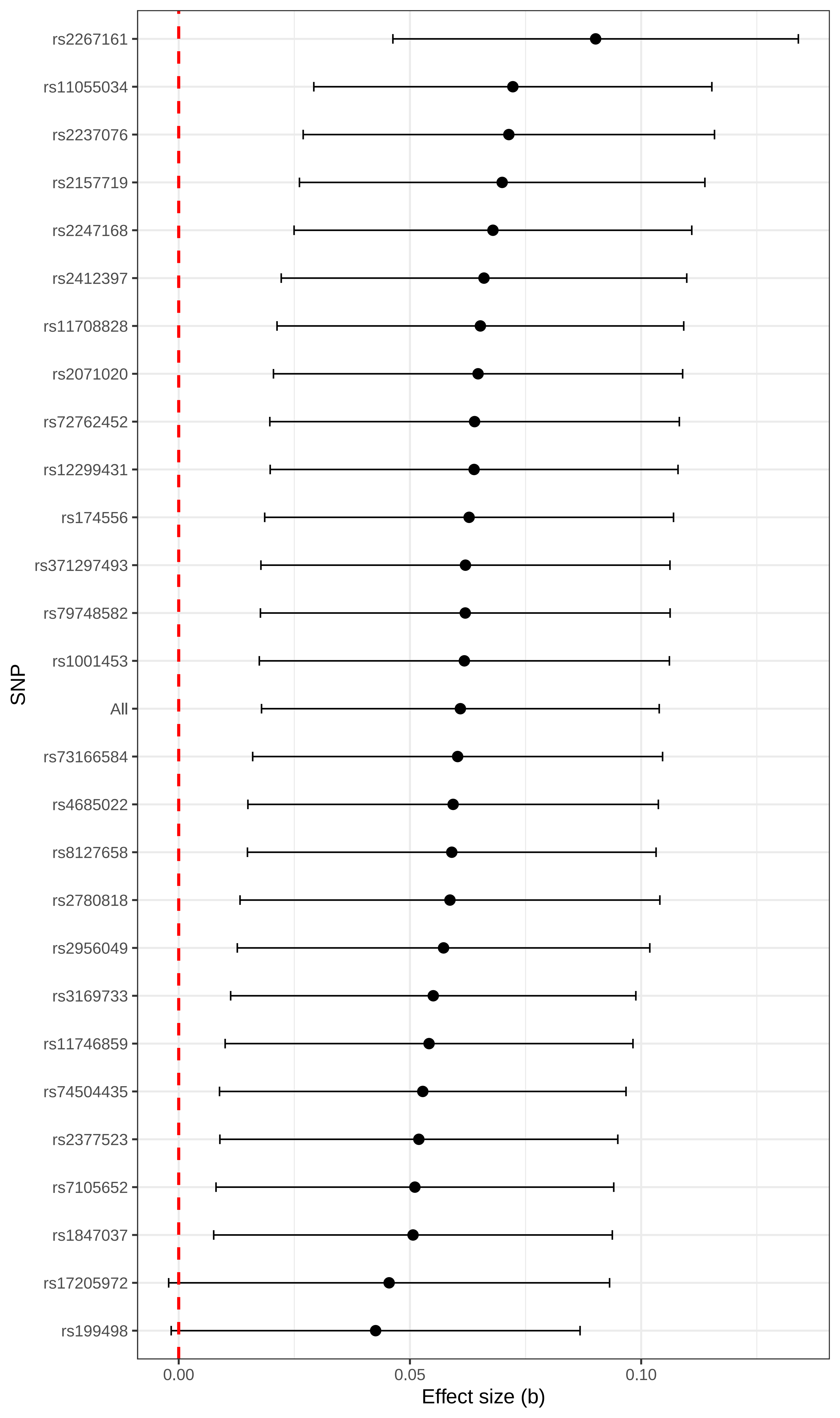

rs2267161
rs11055034
rs2237076
rs2157719
rs2247168
rs2412397
rs11708828
rs2071020
rs72762452
rs12299431
rs174556
rs371297493
rs79748582
rs1001453
All
rs73166584
rs4685022
rs8127658
rs2780818
rs2956049
rs3169733
rs11746859
rs74504435
rs2377523
rs7105652
rs1847037
rs17205972
rs199498
SNP
0.00
0.05
0.10
Effect size (b)
